# Association of DNA methylation signatures with premature ageing and cardiovascular death in patients with end-stage kidney disease: a pilot epigenome-wide association study

**DOI:** 10.1080/15592294.2023.2214394

**Published:** 2023-05-19

**Authors:** Keiichi Sumida, Khyobeni Mozhui, Xiaoyu Liang, Yamini Mallisetty, Zhongji Han, Csaba P. Kovesdy

**Affiliations:** a Division of Nephrology, Department of Medicine, University of Tennessee Health Science Center, Memphis, TN, USA; b Department of Preventive Medicine, University of Tennessee Health Science Center, Memphis, TN, USA; c Department of Epidemiology and Biostatistics, Michigan State University College of Human Medicine, East Lansing, MI, USA

**Keywords:** Aging, cardiovascular disease, DNA methylation, epigenetics clock, end-stage kidney disease, mortality

## Abstract

Patients with end-stage kidney disease (ESKD) display features of premature aging. There is strong evidence that changes in DNA methylation (DNAm) contribute to age-related pathologies; however, little is known about their association with premature aging and cardiovascular mortality in patients with ESKD. We assayed genome-wide DNAm in a pilot case-control study of 60 hemodialysis patients with (n=30, cases) and without (n=30, controls) a fatal cardiovascular event. DNAm was profiled on the Illumina EPIC BeadChip. Four established DNAm clocks (i.e., Horvath-, Hannum-, Pheno-, and GrimAge) were used to estimate epigenetic age (DNAmAge). Epigenetic age acceleration (EAA) was derived as the residuals of regressing DNAmAge on chronological age (chroAge), and its association with cardiovascular death was examined using multivariable conditional logistic regression. An epigenome-wide association study (EWAS) was performed to identify differentially methylated CpGs associated with cardiovascular death. All clocks performed well at predicting chroAge (correlation between DNAmAges and chroAge of r=0.76-0.89), with GrimAge showing the largest deviation from chroAge (a mean of +21.3 years). There was no significant association of EAAs with cardiovascular death. In the EWAS, a CpG (cg22305782) in the *FBXL19* gene had the strongest association with cardiovascular death with significantly lower DNAm in cases vs. controls (*P*_FDR_=2.0x10^−6^). *FBXL19* is involved in cell apoptosis, inflammation, and adipogenesis. Overall, we observed more accelerated aging in patients with ESKD, although there was no significant association of EAAs with cardiovascular death. EWAS suggests a potential novel DNAm biomarker for premature cardiovascular mortality in ESKD.

## Introduction

End-stage kidney disease (ESKD) is a condition characterized by a disproportionately high risk of cardiovascular morbidity and mortality, which is almost exclusively observed at much younger ages than in the general population [1]. This distinct nature of ESKD closely resembles the phenotypic features of premature biological ageing, which, contrary to chronological ageing, is characterized by progressive loss of physical capability and function, often in combination with accelerated accumulation of so-called ageing-related diseases, including cardiovascular disease [2–4]. Several nutritional, lifestyle, and disease-specific factors have been suggested to contribute to premature biological ageing and cardiovascular mortality in ESKD through complex pathophysiological mechanisms, such as persistent low-grade inflammation, DNA damage, and decreased klotho expression [4,5]. Considerable efforts have been made to improve outcomes in patients with ESKD by targeting known risk factors; however, the substantial disease burden attributable to premature ageing and cardiovascular disease in these patients remains unresolved. Continued efforts are therefore needed to identify novel biomarkers that can reliably estimate biological age and predict the excess risk of premature cardiovascular mortality in patients with ESKD.

DNA methylation (DNAm) is one of the most common epigenetic modifications influenced by several lifestyle and environmental factors. As a key regulator of transcriptional activity, DNAm has been implicated in the onset and progression of complex diseases, including diabetes, chronic kidney disease (CKD), and cardiovascular disease [6–8]. Recently, several ‘epigenetic clocks’ have been developed to estimate epigenetic age (a.k.a. DNAmAge) based primarily on changes in age-dependent DNAm, which could not only reflect individuals’ biological age but also predict ageing-related outcomes [9–15]. Epigenome-wide association studies (EWASs) and related pathway enrichment analysis have also revealed that changes in DNAm signatures play a fundamental role in the pathogenesis of various ageing-related diseases, including cardiovascular disease [16–20]. These observations suggest that changes in DNAm signatures could be critically important in explaining the acceleration of biological ageing and the high rate of premature cardiovascular mortality in patients with ESKD. Despite this plausibility and the potential of DNAm as a novel biomarker for premature cardiovascular mortality in ESKD, data are scarce on changes in DNAm patterns associated with premature ageing and cardiovascular mortality in this relevant population.

We hypothesized that patients with ESKD who died of a cardiovascular event would have a more accelerated DNAmAge compared with those without such an event and that those with (vs. without) a fatal cardiovascular event would display differentially methylated cytosine-phosphate-guanine (CpG) sites, which could be involved in cardiovascular pathophysiology. In this pilot case-control study of patients with ESKD receiving haemodialysis therapy, we therefore aimed to estimate DNAmAge and examine the association of epigenetic age acceleration (EAA) (i.e., the residuals of regressing DNAmAge on chronological age [chroAge]) with cardiovascular death, and also perform an EWAS to identify differentially methylated CpGs associated with cardiovascular death in these patients.

## Materials and methods

### Study design

This was a pilot case-control study sourced from a prospective study of anonymized samples and statistically de-identified clinical data (detailed below) obtained from a biorepository assembled by DaVita Clinical Research (Minneapolis, MN, USA). Anonymized samples and statistically de-identified data were made available to the authors for academic research via a grant program called BioReG.

### Study population

The DaVita Clinical Research biorepository comprises blood samples and clinical data from 4,028 individuals with prevalent end-stage renal disease who received haemodialysis at a large dialysis organization (LDO) between May 2011 and October 2013, as previously described [21]. Patients with haemoglobin <8.0 g/dL, who were <18 years of age, who were pregnant, or who had any physical, mental, or medical condition which prohibited the ability to provide informed consent were excluded from participation. The biorepository sampling protocol was reviewed and approved by an Institutional Review Board (IRB) (Quorum IRB, Seattle, WA, USA) and patients provided written informed consent prior to the initiation of sample collection.

For the present pilot case-control study, we used whole blood samples at baseline (or at the first study visit if baseline samples were not sufficiently available) and clinical data corresponding to the blood sampling date in a total of 60 haemodialysis patients within the repository housed at the University of Tennessee Health Science Center (UTHSC) (UT-DaVita haemodialysis cohort; *n* = 978) [22,23]. Cases (*n* = 30) were haemodialysis patients who died of a cardiovascular event (with a median follow-up of 1.5 years), while controls (*n* = 30) were those who remained alive without developing any cardiovascular events over the entire follow-up (a median of 2.3 years), matched 1:1 by age, sex, race, and dialysis vintage to account for major non-modifiable cardiovascular risk factors. The study was approved by the IRB at UTHSC (IRB protocol numbers: 16–04357-XP and 17–05299-XP).

### Biorepository biospecimen and clinical data collection

Under the biorepository study protocol, blood samples were collected from each subject at baseline and, thereafter, every 3 months for up to one year. Pre-dialysis blood samples were collected and processed according to a standardized protocol as previously described [21–23]. Briefly, anonymized plasma samples were shipped in refrigerated packs from the centralized laboratory to the researchers and stored at −80°C. Clinical data for each biorepository subject were collected by the LDO during the course of routine care and were maintained in the LDO electronic health record. The data were then provided to the researchers by DaVita Clinical Research in a statistically de-identified form. Cardiovascular death was defined as death caused by acute myocardial infarction, atherosclerotic heart disease, cardiomyopathy, cardiac arrhythmia, cardiac arrest, or congestive heart failure [23]. Death due to cerebrovascular disease was not included in the cardiovascular death.

### DNA extraction, quantification, and methylation assessment

Genomic DNA was extracted from whole blood cells using the QIAamp DNA Blood Mini Kit (Qiagen, USA) and quantified using a Qubit fluorometer. DNA methylation was assayed on the Illumina Infinium MethylationEPIC array. Standard manufacturer’s protocol was used, and this was carried out by the Epigenomic Services from Diagenode (Cat nr. G02090000). The genomic DNA was then deaminated with the EZ-96 DNA Methylation Kit (Zymo Research) according to Illumina’s recommended deamination protocol, and the Illumina Infinium MethylationEPIC array BeadChip (850K) was carried out to measure DNAm by the Epigenomic Services from Diagenode (Cat nr. G02090000).

Raw intensity IDAT files were processed using the R package, minfi [24], as described previously [25,26]. In brief, CpG probes with detection *p*-value >0.01, and probes that overlap single-nucleotide polymorphism (SNPs) and/or are flagged for poor mapping quality and cross-reactivity were excluded. A total of 835,424 high-quality probes were retained and used for downstream analyses, and the data were quantile normalized. The beta-values showed the expected bimodal distribution (**Supplementary Figure S1A**). As an additional quality check, we derived the methylation-based imputed sex, and cross-verified imputed sex versus self-reported gender, and found no discrepancy. For outlier detection, we performed a principal component (PC) analysis on the autosomal CpGs (816,126 probes in total), and a plot between PC1 and PC2 showed no outlier sample (**Supplementary Figure S1B**). To account for cellular heterogeneity, we applied a reference-based approach to estimate the proportions of CD4+ T cells, CD8+ T cells, natural killer T cells, B cells, monocytes, and neutrophils [27–29]. Pearson’s correlations between the top PCs and the estimated blood cell proportions showed that PC1 had significant inverse correlations with estimated proportion of B and T lymphocytes, and positive correlation with neutrophils; while PC2 and PC3 had significant positive correlations with both B and CD4+ T lymphocytes and CD4+ T lymphocytes, respectively (**Supplementary Table S1**).

### Statistical analysis

Baseline patient characteristics by cardiovascular case status were presented as numbers (percentages) for categorical variables and mean (standard deviation [SD]) for continuous variables with a normal distribution or median (interquartile interval [IQI]) for those with a skewed distribution. Variables with a skewed distribution were treated as log-transformed continuous variables, as appropriate. Differences between groups were assessed using Fisher’s exact test, t-test, or Wilcoxon rank-sum test, as appropriate.

### DNAmAge and epigenetic age acceleration

DNAmAge was estimated using four established algorithms by Horvath et al. [10], Hannum et al. [9], Levine et al. [30], and Lu et al. [31] based primarily on their age-prediction ability and strong associations with mortality. For this, we submitted our DNAm data to the DNAmAge calculator (https://dnamage.genetics.ucla.edu/home) developed by Horvath et al. [10] The EAA for each estimated DNAmAge (i.e., Horvath-, Hannum-, Pheno-, and GrimAge) was then calculated based on the residuals of regressing DNAmAge on chroAge [32,33]. The correlation between each DNAmAge and chroAge was assessed using Pearson’s correlation coefficient. We then compared the EAAs between cases and controls. In order to examine the risk of cardiovascular death associated with each EAA, we applied multivariable conditional logistic regression models. Given the limited sample size of this pilot study, the exposure was treated as a continuous variable, and the following incremental models were used to account for potential confounders on the basis of theoretical consideration and data availability: model 1 was unadjusted; model 2 included age and dialysis vintage (i.e., length of time on dialysis treatment) to account for the residual imbalance of these continuous matching factors; and model 3 was additionally adjusted for estimated cell proportions (i.e., CD4+ T cells, CD8+ T cells, natural killer T cells, B cells, monocytes, and neutrophils). Because no between-group dissimilarity was observed for diabetes, a key potential confounder, as well as categorical matching factors (i.e., sex and race), these variables were not accounted for in this analysis. As a sensitivity analysis, the association analysis was repeated for intrinsic EAA (IEAA), which measures ‘pure’ epigenetic ageing effects that are not confounded by differences in blood cell counts [34,35]. All analyses were performed in patients with complete data available using STATA/MP Version 17 (STATA Corporation, College Station, TX). A threshold of statistical significance was set at the level of *P* < 0.05 for these association analyses.

### Epigenome-wide association analysis

An EWAS was performed to identify differentially methylated CpG sites associated with cardiovascular death with adjustment for relevant variables. This was performed on R using the regression model CpG ~ status + age + sex + self-reported race/ethnicity + top 5 PCs, where status is whether the individual had cardiovascular death or not. We included the top PCs because these capture the cell heterogeneity and possibly variance from other unmeasured variance. To define CpGs that are associated with cardiovascular death, genome-wide significance threshold was set at 9e-08, and the suggestive threshold was set at 5e-05 [36]. The quantile-quantile (QQ) and Manhattan plots were generated using the R package, QQman [37]. To evaluate the robustness of most significant CpG sites associated with cardiovascular death, we repeated an EWAS by replacing the top 5 PCs with estimated cell proportions (i.e., CD4+ T cells, CD8+ T cells, natural killer T cells, B cells, monocytes, and neutrophils) in the adjustment as a sensitivity analysis.

## Results

### Baseline characteristics

Patients’ baseline characteristics by cardiovascular case status are presented in Table 1. Cases and controls were of similar age at baseline (means of 63.2 ± 11.4 and 63.0 ± 11.7 years, respectively) and by design did not differ for other matching factors, including sex (60.0% male in both), self-reported race/ethnicity (53.4% African American in both), and dialysis vintage (4.4 ± 3.6 and 4.5 ± 3.1 years, respectively). Compared with controls, cases were less likely to use a dialysis catheter and tended to have higher systolic and diastolic blood pressures and higher prevalence of erythropoiesis stimulating agent use, although none of the differences reached statistical significance.

**Table 1.** Baseline patient characteristics by cardiovascular case status.

Characteristics	Cases (*n* = 30)	Controls^a^ (*n* = 30)	*P*
Age (years)	63.2 ± 11.4	63.0 ± 11.7	0.95
Male sex	18 (60.0)	18 (60.0)	1.00
Race			1.00
White	13 (43.3)	13 (43.3)	
African American	16 (53.4)	16 (53.4)	
Others	1 (3.3)	1 (3.3)	
Dialysis vintage (years)	4.4 ± 3.6	4.5 ± 3.1	0.90
Vascular access type			0.35
Arteriovenous fistula	20 (66.7)	15 (50.0)	
Arteriovenous graft	6 (20.0)	7 (23.3)	
Catheter	4 (13.3)	8 (26.7)	
Cause of ESKD			0.53
Diabetes mellitus	17 (56.7)	15 (50.0)	
Hypertension	6 (20.0)	10 (33.3)	
Others	7 (23.3)	5 (16.7)	
Body mass index (kg/m^2^)	30.6 ± 8.0	30.6 ± 7.3	1.00
Systolic BP (mmHg)	145.4 ± 26.1	156.5 ± 25.9	0.11
Diastolic BP (mmHg)	74.7 ± 15.3	79.4 ± 18.1	0.29
Charlson Comorbidity Index	5.8 ± 1.8	6.1 ± 1.6	0.60
Comorbidities			
Diabetes mellitus	21 (70.0)	21 (70.0)	1.00
Ischemic heart disease	5 (16.7)	4 (13.3)	0.72
Congestive heart failure	6 (20.0)	3 (10.0)	0.28
Liver disease	1 (3.3)	2 (6.7)	0.55
HIV/AIDS	0 (0)	0 (0)	1.00
Malignancies	0 (0)	0 (0)	1.00
Laboratory parameters			
Blood hemoglobin (g/dL)	11.0 ± 1.3	10.9 ± 0.8	0.59
Serum albumin (g/dL)	4.0 ± 0.3	3.9 ± 0.3	0.22
Serum calcium (mg/dL)	9.3 ± 0.7	9.1 ± 0.7	0.27
Serum phosphorus (mg/dL)	5.7 ± 1.5	5.2 ± 1.2	0.68
Serum ALP (U/L)	114.6 ± 51.3	104.6 ± 43.0	0.42
Serum intact PTH (pg/mL)	342 [226, 708]	339 [219, 504]	0.08
Medications			
Statins	7 (23.3)	6 (20.0)	0.75
ESAs	21 (70.0)	27 (90.0)	0.053
Phosphate binders	17 (56.7)	20 (66.7)	0.43
Vitamin D analogs	27 (90.0)	28 (93.3)	0.64
Aspirin	6 (20.0)	5 (16.7)	0.74
Opioids	12 (40.0)	11 (36.7)	0.79
Antibiotics	0 (0)	0 (0)	1.00
Infectious hospitalization	0 (0)	0 (0)	1.00
Culture positive bacteremia	0 (0)	0 (0)	1.00

Data are presented as number (percentage), mean ± SD, or median [interquartile interval].

^a^Matched by age, sex, race, and dialysis vintage.

Abbreviations: ALP = alkaline phosphatase; BP = blood pressure; ESAs = erythropoiesis-stimulating agents; ESKD = end-stage kidney disease; HIV/AIDS = human immunodeficiency virus/acquired immunodeficiency syndrome; PTH = parathyroid hormone.

### DNAmage, epigenetic age acceleration, and cardiovascular death

**Figure 1.**
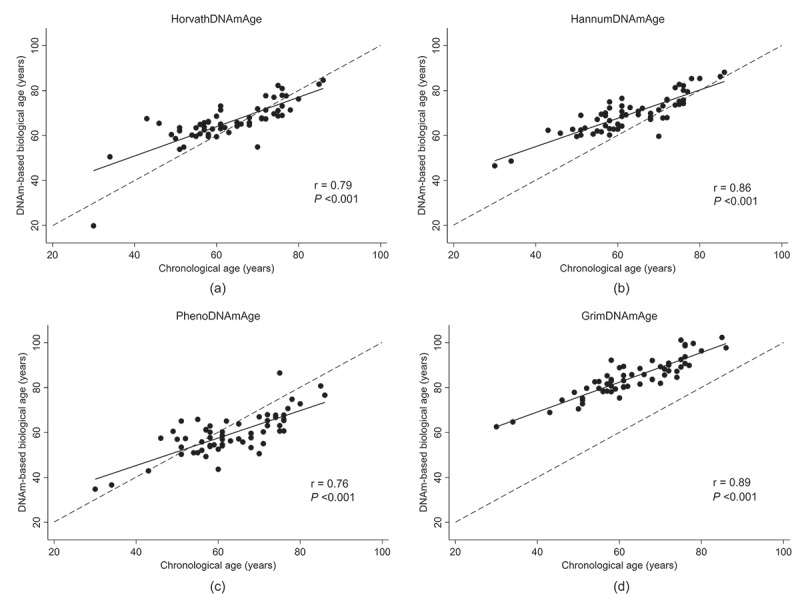
Correlation between chronological age and DNA methylation-based biological age according to four established epigenetic clocks in 60 hemodialysis patients; (A) HorvathAge, (B) HannumAge, (C) PhenoAge, and (D) GrimAge.

*Note*: The solid line represents least-squares linear regression line, while the dashed line represents the perfect correlation between chronological and biological age.

**Figure 2.**
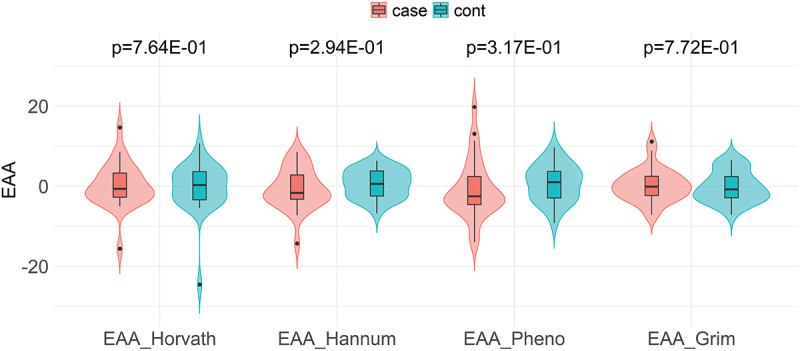
Violin plots comparing epigenetic age accelerations between cardiovascular cases and controls* according to Horvath-, Hannum-, Pheno-, and GrimAge.

As depicted in Figure 1, there was a significant positive and strong correlation between DNAmAge and chroAge for all four epigenetic clocks; i.e., Horvath- (*r* = 0.79, *P* < 0.001), Hannum- (*r* = 0.86, *P* < 0.001), PhenoAge (*r* = 0.76, *P* < 0.001), and GrimAge (*r* = 0.89, *P* < 0.001). Among these, GrimAge had the largest deviation from chroAge with a mean (95% confidence interval [CI]) deviation of +21.3 (+19.9 to +22.8) years (Figure 1d). Although the deviation from chroAge for the other three DNAmAges was much smaller than that of GrimAge, Horvath- and HannumAge had positive deviations from chroAge (a mean [95% CI] of +2.9 [+1.1 to +4.7] and +6.5 [+5.0 to +8.1] years, respectively; Figure 1a,b), while a negative deviation from chroAge was observed for PhenoAge (a mean [95% CI] of −3.7 [−5.6 to −1.7] years; Figure 1c). When comparing the EAA for each epigenetic clock (Horvath-, Hannum-, Pheno-, or GrimAge) between cases and controls, no significant difference was observed for all four epigenetic clocks (Figure 2). **Supplementary Table S2** shows the association of EAAs with cardiovascular death, using univariable and multivariable conditional logistic regression analyses. In the univariable model, none of the four EAAs were significantly associated with cardiovascular death. These EAA-cardiovascular death associations remained non-significant after adjustment for age, dialysis vintage, and estimated cell proportions (i.e., CD4+ T cells, CD8+ T cells, natural killer T cells, B cells, monocytes, and neutrophils). Similarly, there was no significant association between IEAA and cardiovascular death (**Supplementary Table S2**).

*Matched by age, sex, race, and dialysis vintage.

*Note*: None of the between-group differences were statistically significant.

### Epigenome-wide association study

**Figure 3.**
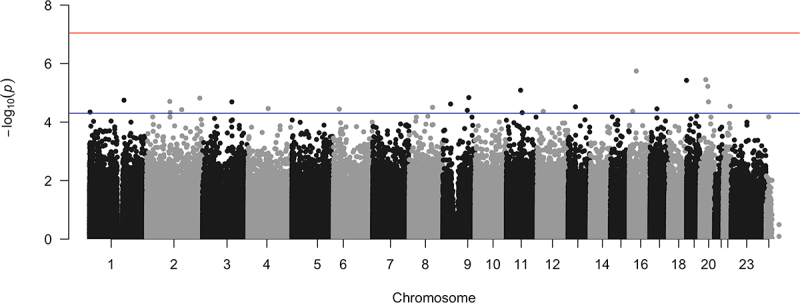
Manhattan plot of the association between DNA methylation and cardiovascular death in 60 hemodialysis patients.

*Note*: The upper (red) and lower (blue) horizontal solid lines represent the genome-wide significance threshold (set at *p*-value of 9.0x10^−8^) and suggestive threshold (set at *p*-value of 5.0x10^−5^).

As depicted in the Manhattan plots (Figure 3), there were a total of 25 CpGs that were above the genome-wide suggestive threshold (at *p*-value of 5.0 × 10^−5^), although the QQ plot and *p*-value distribution did not provide evidence of a significant association between DNAm and cardiovascular death (**Supplementary Figure S2**). Among these, the top 10 most significant CpG sites associated with cardiovascular death (i.e., cases vs. controls) are listed in Table 2.

All but three CpG sites (i.e., cg08842907, cg25129798, and cg04578023) showed significantly lower DNAm (i.e., hypomethylated) in cases vs. controls. The top CpG site that had the strongest association with cardiovascular death was cg22305782 (chr 16:30937214, *p*-value = 2.0×10^−6^) located within the *FBXL19* (F-Box And Leucine Rich Repeat Protein 19) gene, which is involved in inflammation, apoptosis, and cell migration [38–40]. None of these top 10 most significant CpG sites associated with cardiovascular death were present among the CpGs underlying the Horvath-, Hannum-, and PhenoAge, which are publicly available.

**Table 2.** Top 10 most significant CpG sites associated with cardiovascular death based on the genome-wide suggestive threshold at *p*-value of 5.0 × 10^−5^.

	Probe no.	Gene symbol	Chr	Position	Status estimate*	*p*-value
1	cg22305782	*FBXL19*	16	30937214	−0.135	2.00E–06
2	cg03111498	*VSX1*	20	25062860	−0.258	4.00E–06
3	cg19558049	*MIER2*	19	306297	−0.178	4.00E–06
4	cg15711208	*MMP24*	20	33836573	−0.258	6.00E–06
5	cg08842907	*BSCL2*	11	62470818	0.193	8.00E–06
6	cg03874948	-	9	113361270	−0.105	1.00E–05
7	cg25129798	-	2	231189493	0.132	2.00E–05
8	cg05431687	*MCL1*	1	150552130	−0.217	2.00E–05
9	cg17273588	*AFF3*	2	100760351	−0.107	2.00E–05
10	cg04578023	*TGM2*	20	36761479	0.140	2.00E–05

*Difference in methylation M-value between cases vs. controls (reference).

The epigenome-wide association study (EWAS) was performed using the following regression model: CpG ~ status + age + sex + self-reported race/ethnicity + top 5 principal components.

When we repeated an EWAS by replacing the top 5 PCs in the adjustment with estimated cell proportions (i.e., CD4+ T cells, CD8+ T cells, natural killer T cells, B cells, monocytes, and neutrophils), the aforementioned top 10 most significant CpG sites (in Table 2) were no longer observed among the top 10 significant CpG sites in this sensitivity EWAS with one exception (**Supplementary Table** **3**). A CpG (cg19558049, chr 19:306297, *p*-value of 6.0 × 10^−6^) located in the *MEIR2* (Mesoderm Induction Early Response 1, Family Member 2) gene remained within the top 10 most significant CpG site associated with cardiovascular death. This gene is involved in the recruitment of histone deacetylase complexes [41].

## Discussion

In this pilot case-control study of patients with ESKD receiving maintenance haemodialysis, we found that four established DNAm clocks (i.e., Horvath-, Hannum-, Pheno-, and GrimAge) performed well at predicting chroAge (*r* = 0.76–0.89), with GrimAge having the strongest correlation with chroAge and showing the largest deviation from chroAge (+21.3 years); although none of the four EAAs were significantly associated with cardiovascular death. In the EWAS, we identified a CpG site (cg22305782, mapping to the *FBXL19* gene) that had the strongest association with cardiovascular death. Furthermore, a sensitivity EWAS exploration identified a common CpG site (cg19558049, mapping to the *MEIR2* gene) that remained among the top 10 CpG sites associated with cardiovascular death.

Increased allostatic load as a result of inflammation, oxidative stress, and uraemic toxins is a hallmark of the uraemic milieu and has been linked to premature cellular ageing, senescence, and apoptosis, i.e., the processes leading to premature vascular calcification and cardiovascular disease in patients with ESKD [22,42,43]. As part of various environmental stimuli that alter epigenetic mechanisms [44], this allostatic overload in uraemic milieu has been suggested to modulate the genomic activities through alterations of DNAm signatures and contribute to inflammatory and/or proatherogenic processes that lead to the development of various ageing-related diseases, including cardiovascular disease [45–47]. In a previous case-control study of 10 ESKD patients undergoing haemodialysis and 10 healthy age- and sex-matched controls, researchers performed a genome-wide analysis of DNAm in peripheral blood mononuclear cells using SuperTAG methylation-specific digital karyotyping and identified several candidate genes differentially methylated in ESKD patients vs. controls [46]. They also classified the differentially methylated genes into distinct proatherogenic processes, including lipid metabolism and transport, cell proliferation and cell-cycle regulation, angiogenesis, and inflammation [46]. In another longitudinal cohort study of patients with CKD and ESKD, researchers quantitated the levels of DNAm in peripheral blood leucocytes using a Luminometric Methylation Assay [48], and demonstrated that the global DNA hypermethylation status (defined as the *Hpa*II/*Msp*I ratio <median) was significantly associated with a higher risk of cardiovascular death among patients who started dialysis therapy, independent of chroAge, inflammation, and history of diabetes mellitus and cardiovascular disease [45]. These two studies, however, are limited in scalability to capture the genome-wide CpG data compared with the newest Illumina EPIC BeadChip array, which can interrogate over 850,000 methylation sites and allows better coverage of CpG sites across the genome [49]. Furthermore, previous studies in patients with ESKD were not designed to identify CpGs associated with cardiovascular death in this relevant population. In the present study, we therefore used a matched case-control cohort design and performed comprehensive profiles of DNAm using the state-of-the-art Illumina EPIC BeadChip array to identify CpGs significantly associated with cardiovascular death in patients with ESKD. Also of note, to our knowledge, we are the first to report DNAmAges estimated using established epigenetic clocks in patients with ESKD [50], along with the most remarkable deviation of GrimAge (vs. Horvath-, Hannum-, and PhenoAge) from chroAge in this population.

GrimAge is a linear combination of chroAge, sex, and 1,030 CpG sites modelled as surrogate biomarkers for seven plasma proteins and smoking pack-years [31]. This GrimAge is not only associated with blood glucose-, insulin-, and triglyceride levels, anthropometric measures of adiposity (i.e., body mass index and waist-to-hip ratio), and hypertension and type 2 diabetes status, but also highly predictive of lifespan and incident cardiovascular disease, which outperforms the other three established DNAmAges [31]. Given the strong association of ESKD with GrimAge-associated clinical factors, it was conceivable that GrimAge (vs. other DNAmAges) had the strongest correlation with chroAge in our study; but it was surprising to see such a large deviation of GrimAge from chroAge (a mean of +21.3 years). Although no significant association was observed between GrimAge-based EAA and cardiovascular death, the observed large deviation of GrimAge from chroAge may reflect more accelerated biological ageing in patients with (vs. without) ESKD and thereby help provide important biological and prognostic information beyond chroAge in this patient population, which deserves future investigation in a larger cohort including other DNAm clocks reported to date.

Another notable finding of this study was the identification of hypomethylated CpG sites significantly and strongly associated with cardiovascular death, namely, cg22305782 and cg19558049 that mapped to *FBXL19* and *MIER2* genes, respectively. *FBXL19* gene encodes a member of the Skp1-Cullin-F-box family of E3 ubiquitin ligases and has been shown to be involved in diverse cellular functions including proliferation, migration, apoptosis, and immune responses [38–40]. More recent work has highlighted a contribution of *FBXL19* to adipogenic properties [51]. Meanwhile, the *MIER2* gene regulates gene transcription via the recruitment of histone deacetylase complexes [41], which has been implicated in multiple processes pertinent to cardiometabolic diseases, including cardiac hypertrophy and remodelling, fibrosis, calcium handling, inflammation and energy metabolism [52]. Although the direct impact of *FBXL19* and *MIER2* genes on cardiovascular disease remain unclear, given the close biological link of the aforementioned cellular and molecular functions to the pathogenesis of cardiometabolic disease [53], the CpG hypomethylation in the *FBXL19* and *MIER2* genes in patients with ESKD might play a distinct mechanistic role in their premature cardiovascular death.

The study results must be interpreted in light of several limitations. Given the substantial heterogeneity of haemodialysis patients with various aetiologies and comorbidities, our study population was not representative of all patients with ESKD. Our biorepository cohort consisted only of patients with ESKD on haemodialysis, and hence the results are not generalizable to CKD patients not on dialysis. Although we adjusted for reference-based estimates of cellular proportions in the analyses, the potential heterogeneity in methylome signal attributable to the underlying cellular heterogeneity cannot be fully eliminated. Due to the small sample size and limited data availability of this pilot case-control study, we were unable to fully account for potential confounders (e.g., smoking, alcohol intake, waist size, body fat, C-reactive protein, triglycerides, and childhood obesity) despite matching and were also likely underpowered to detect a significant association of EAAs with cardiovascular death (i.e., type II error is possible, **Supplementary Data**). Lastly, the present study could not address potential temporal epigenetic changes and their associations with outcomes, which however is beyond the scope of this pilot case-control study and needs to be examined in future large-scale longitudinal studies along with comprehensive pathway analysis.

In conclusion, in this pilot case-control study of prevalent haemodialysis patients, we estimated DNAmAge using four established epigenetic clocks and observed more accelerated ageing in the ESKD population. In addition, by performing an EWAS, we identified hypomethylated CpG sites that were significantly associated with cardiovascular death in ESKD. Although these findings should be validated in more in-depth and larger studies, our preliminary results provide novel insights into premature biological ageing in patients with ESKD and suggest a potential novel DNAm biomarker for premature cardiovascular death in these patients.

## Supplementary Material

Supplemental Material

## Data Availability

The data used in these analyses were provided by DaVita Clinical Research. Requests for access to data can be made in writing to DaVita Clinical Research.
