# Supplementary material for: Association of DNA methylation signatures with premature ageing and cardiovascular death in patients with end-stage kidney disease: a pilot epigenome-wide association study
Source: Epigenetics. 2023 May 19;18(1):2214394. doi: 10.1080/15592294.2023.2214394 (PMC10202091; doi:10.1080/15592294.2023.2214394)
Supplement: Supplemental Material [file KEPI_A_2214394_SM2873.docx]

**SUPPLEMENTARY MATERIALS**

**Supplementary Table 1**. Pearson’s correlations between the top PCs and the estimated blood cell proportions

**Supplementary Table 2**. Odds ratios and 95% confidence interval for cardiovascular death associated with EAAs based on four established epigenetic clocks and IEAA in 60 hemodialysis patients

**Supplementary Table 3**. Top 10 most significant CpG sites associated with cardiovascular death based on the genome-wide suggestive threshold at *p*-value of 5.0x10^-5^ after replacing the top 5 principal components with estimated cell proportions (i.e., CD4+ T cells, CD8+ T cells, natural killer T cells, B cells, monocytes, and neutrophils) in the EWAS regression model

**Supplementary Figure 1**. Summary data on quality control of DNA methylation assay: (A) density plot of beta-value and (B) quality control plot

**Supplementary Figure 2**. EWAS report of (A) QQ plot and (B) *p*-value distribution

**Supplementary data**. A power analysis assessing the effect size to be detectable in this pilot study

**Supplementary Table 1**. Pearson’s correlations between the top PCs and the estimated blood cell proportions

| **Variable** | **by Variable** | **Correlation** | **Count** | **Lower 95%** | **Upper 95%** | **P value** |
| --- | --- | --- | --- | --- | --- | --- |
| PC1 | counts.Bcell | -0.46 | 60 | -0.6365 | -0.229 | 0.0002 |
|  | counts.CD4T | -0.39 | 60 | -0.5831 | -0.1468 | 0.0023 |
|  | counts.CD8T | -0.79 | 60 | -0.8706 | -0.6731 | 0.00001 |
|  | counts.Mono | -0.11 | 60 | -0.3545 | 0.1476 | 0.4006 |
|  | counts.Neu | 0.90 | 60 | 0.8316 | 0.937 | 0.00001 |
|  | counts.NK | -0.42 | 60 | -0.6109 | -0.1889 | 0.0008 |
| PC2 | counts.Bcell | 0.40 | 60 | 0.1574 | 0.5901 | 0.0018 |
|  | counts.CD4T | 0.48 | 60 | 0.2596 | 0.6555 | 0.00001 |
|  | counts.CD8T | -0.30 | 60 | -0.5139 | -0.0488 | 0.0203 |
|  | counts.Mono | 0.08 | 60 | -0.1743 | 0.3303 | 0.5267 |
|  | counts.Neu | -0.19 | 60 | -0.4261 | 0.0639 | 0.1393 |
|  | counts.NK | -0.24 | 60 | -0.4621 | 0.0192 | 0.0696 |
| PC3 | counts.Bcell | 0.09 | 60 | -0.1636 | 0.34 | 0.474 |
|  | counts.CD4T | 0.60 | 60 | 0.4068 | 0.7402 | 0.00001 |
|  | counts.CD8T | -0.24 | 60 | -0.4661 | 0.0141 | 0.064 |
|  | counts.Mono | 0.12 | 60 | -0.1389 | 0.3621 | 0.3644 |
|  | counts.Neu | -0.23 | 60 | -0.4601 | 0.0217 | 0.0726 |
|  | counts.NK | -0.14 | 60 | -0.3832 | 0.1149 | 0.2752 |
| PC4 | counts.Bcell | 0.06 | 60 | -0.1933 | 0.3126 | 0.6284 |
|  | counts.CD4T | 0.02 | 60 | -0.2349 | 0.2727 | 0.8782 |
|  | counts.CD8T | -0.05 | 60 | -0.3017 | 0.2049 | 0.6948 |
|  | counts.Mono | 0.29 | 60 | 0.0405 | 0.5078 | 0.0239 |
|  | counts.Neu | -0.07 | 60 | -0.3191 | 0.1863 | 0.59 |
|  | counts.NK | -0.11 | 60 | -0.358 | 0.1436 | 0.3836 |
| PC5 | counts.Bcell | -0.13 | 60 | -0.3729 | 0.1267 | 0.3167 |
|  | counts.CD4T | -0.09 | 60 | -0.3319 | 0.1725 | 0.5177 |
|  | counts.CD8T | 0.06 | 60 | -0.1951 | 0.3109 | 0.6385 |
|  | counts.Mono | -0.19 | 60 | -0.4217 | 0.0694 | 0.1507 |
|  | counts.Neu | 0.15 | 60 | -0.1033 | 0.3931 | 0.2381 |
|  | counts.NK | -0.14 | 60 | -0.3799 | 0.1187 | 0.2879 |

Abbreviations: Mono = monocyte; Neu = neutrophil; NK = natural killer cell; PC = principal component

**Supplementary Table 2**. Odds ratios and 95% confidence interval for cardiovascular death associated with EAAs based on four established epigenetic clocks and IEAA in 60 hemodialysis patients

| **Characteristics** | **Model 1** | | **Model 2** | | **Model 3** | |
| --- | --- | --- | --- | --- | --- | --- |
|  | **OR (95% CI)** | ***P*** | **OR (95% CI)** | ***P*** | **OR (95% CI)** | ***P*** |
| EAA_Horvath_ (per unit increase) | 1.02 (0.92-1.12) | 0.75 | 1.01 (0.91-1.12) | 0.87 | 1.04 (0.92-1.18) | 0.54 |
| EAA_Hannum_ (per unit increase) | 0.91 (0.79-1.06) | 0.23 | 0.89 (0.75-1.04) | 0.15 | 0.86 (0.71-1.05) | 0.14 |
| EAA_Pheno_ (per unit increase) | 0.95 (0.86-1.05) | 0.30 | 0.94 (0.85-1.04) | 0.24 | 0.91 (0.77-1.07) | 0.26 |
| EAA_Grim_ (per unit increase) | 1.03 (0.88-1.21) | 0.72 | 1.02 (0.85-1.22) | 0.86 | 1.06 (0.80-1.41) | 0.67 |
| IEAA (per unit increase) | 1.03 (0.93-1.23) | 0.58 | 1.02 (0.93-1.13) | 0.66 | 1.07 (0.93-1.22) | 0.34 |

*Note*: All models matched for age, sex, race, and dialysis vintage. Model 1 is unadjusted: model 2 is adjusted for age and dialysis vintage to account for residual imbalance; and model 3 is adjusted for the variables in model 2 plus estimated cell proportions (i.e., CD4+ T cells, CD8+ T cells, natural killer T cells, B cells, monocytes, and neutrophils).

Abbreviation: EAA = epigenetic age acceleration; IEAA = intrinsic epigenetic age acceleration

**Supplementary Table 3**. Top 10 most significant CpG sites associated with cardiovascular death based on the genome-wide suggestive threshold at *p*-value of 5.0x10^-5^ after replacing the top 5 principal components with estimated cell proportions (i.e., CD4+ T cells, CD8+ T cells, natural killer T cells, B cells, monocytes, and neutrophils) in the EWAS regression model

|  | Probe no. | Gene symbol | Chr | Position | Status estimate* | *p*-value |
| --- | --- | --- | --- | --- | --- | --- |
| 1 | cg21744150 | *WLS* | 1 | 68687848 | -0.363 | 6.00E-06 |
| 2 | cg19558049 | *MIER2* | 19 | 306297 | -0.177 | 6.00E-06 |
| 3 | cg03882324 | *-* | 1 | 210425800 | -0.270 | 1.00E-05 |
| 4 | cg10760023 | *LILRB2* | 19 | 54784354 | -0.180 | 1.00E-05 |
| 5 | cg27079726 | *PKDREJ* | 22 | 46659317 | 0.175 | 2.00E-05 |
| 6 | cg07486732 | *ASB18* | 2 | 237150793 | -0.138 | 2.00E-05 |
| 7 | cg03042666 | *HDAC4* | 2 | 240014781 | -0.197 | 2.00E-05 |
| 8 | cg11226480 | *LETMD1* | 12 | 51442072 | -0.409 | 2.00E-05 |
| 9 | cg24590990 | *TRPM8* | 2 | 234881435 | 0.176 | 2.00E-05 |
| 10 | cg17429065 | *ACOT7* | 1 | 6453910 | -0.167 | 3.00E-05 |

*Difference in methylation M-value between cases vs. controls (reference)

*Note*: The EWAS was performed using the following regression model: CpG ~ status + age + sex + self-reported race/ethnicity + estimated cell proportions (i.e., CD4+ T cells, CD8+ T cells, natural killer T cells, B cells, monocytes, and neutrophils)

Abbreviation: EWAS = epigenome-wide association study

**Supplementary Figure 1**. Summary data on quality control of DNA methylation assay: (A) density plot of beta-value and (B) quality control plot

(A) (B)


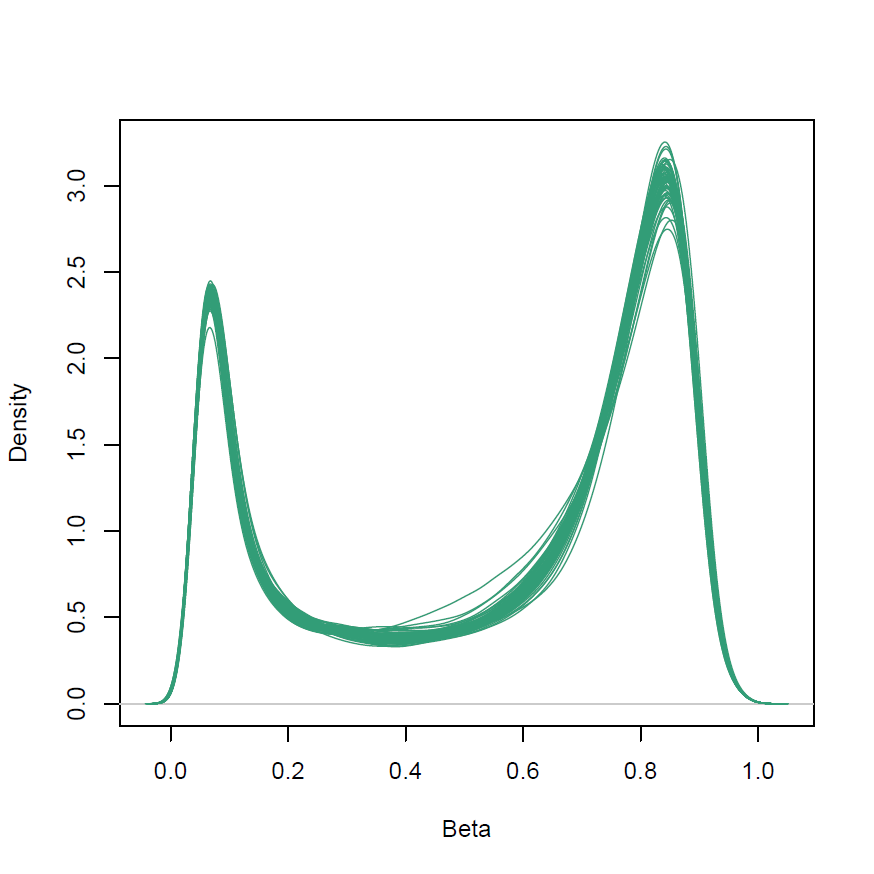

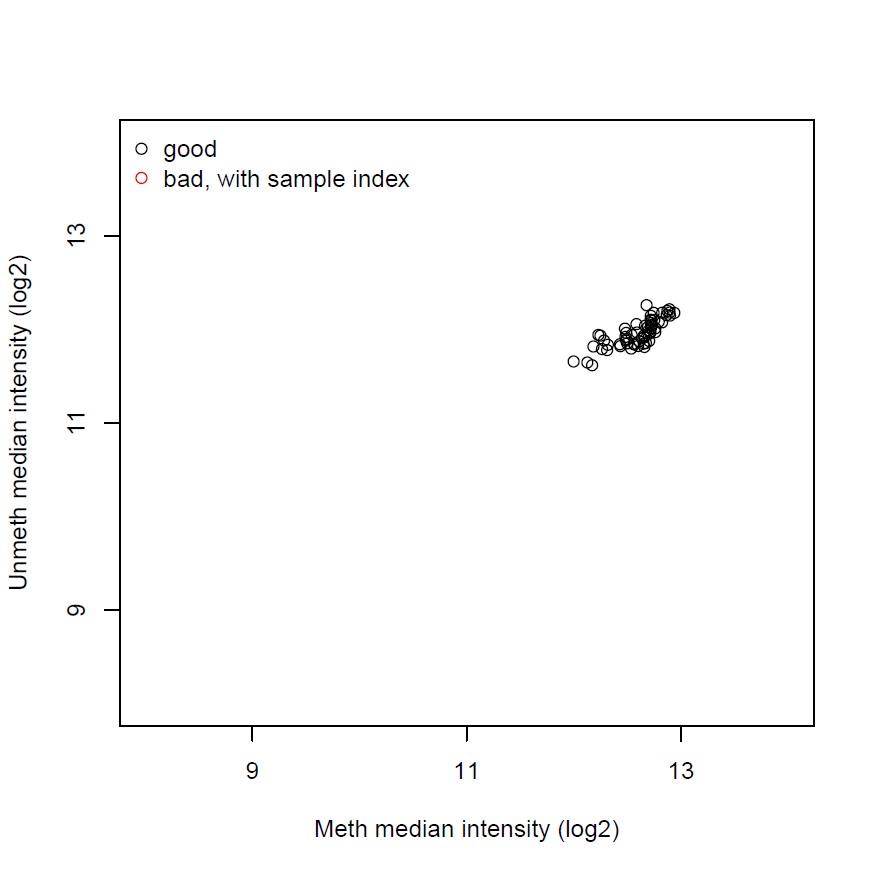


**Supplementary Figure 2**. EWAS report of (A) QQ plot and (B) *p*-value distribution

(A) (B)


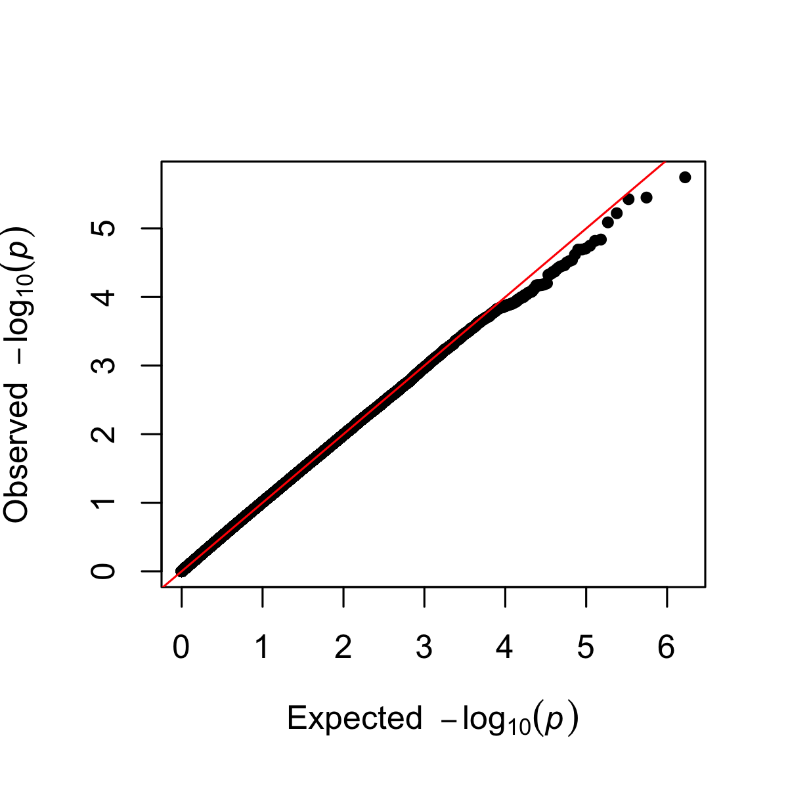

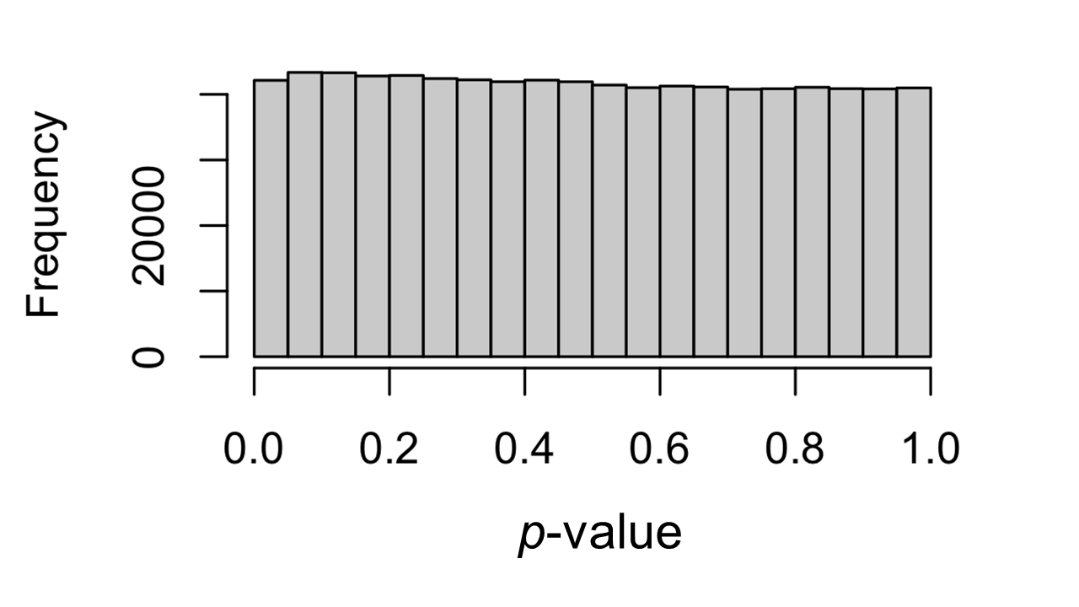


Abbreviations: EWAS = epigenome-wide association study; QQ = quantile-quantile

**Supplementary data**. A power analysis assessing the effect size to be detectable in this pilot study

In order to achieve 80% power at a 0.05 significance level, the sample size of 60 can detect a minimum lower and upper effect sizes (odds ratios) of 0.34 and 2.93 (STATA “power mcc”), respectively, using conditional logistic regression in a matched case-control study.
